# Flowers in the Attic: Lateralization of the detection of meaning in visual noise

**DOI:** 10.1167/jov.20.10.11

**Published:** 2020-10-07

**Authors:** Simon J. Cropper, Ashlan McCauley, O. Scott Gwinn, Megan Bartlett, Michael E. R. Nicholls

**Affiliations:** Melbourne School of Psychological Sciences, University of Melbourne, Melbourne, Australia; School of Psychology, Flinders University, Adelaide, Australia

**Keywords:** vision, pareidolia, noise, signal detection theory, lateralization, Diffusion Decision model, psychophysics

## Abstract

The brain is a slave to sense; we see and hear things that are not there and engage in ongoing correction of these illusory experiences, commonly termed *pareidolia*. The current study investigates whether the predisposition to see meaning in noise is lateralized to one hemisphere or the other and how this predisposition to visual false-alarms is related to personality. Stimuli consisted of images of faces or flowers embedded in pink (1/f) noise generated through a novel process and presented in a divided-field paradigm. Right-handed undergraduates participated in a forced-choice signal-detection task where they determined whether a face or flower signal was present in a single-interval trial. Experiment 1 involved an equal ratio of signal-to-noise trials; experiment 2 provided more potential for illusionary perception with 25% signal and 75% noise trials. There was no asymmetry in the ability to discriminate signal from noise trials (measured using d′) for either faces and flowers, although the response criterion (c) suggested a stronger predisposition to visual false alarms in the right visual field, and this was negatively correlated to the unusual experiences dimension of schizotypy. Counter to expectations, changing the signal-image to noise-image proportion in Experiment 2 did not change the number of false alarms for either faces and flowers, although a stronger bias was seen to the right visual field; sensitivity remained the same in both hemifields but there was a moderate positive correlation between cognitive disorganization and the bias (c) for “flower” judgements. Overall, these results were consistent with a rapid evidence-accumulation process of the kind described by a diffusion decision model mediating the task lateralized to the left-hemisphere.

## Introduction

Humans have a remarkable ability to detect meaningful patterns in visual stimuli, with a particular predilection toward seeing faces whether one is actually present or not: seeing a face in the clouds, a man on the moon, or Jesus on a piece of toast are common archetypes of hallucinations of faces, a phenomenon termed face-pareidolia (Liu, Li, Feng, Li, Tian, & Lee, 2014; Partos, Cropper, & Rawlings, 2016; Rieth, Lee, Lui, Tian, & Huber, 2011). Following on from our previous study (Partos et al., 2016), the work presented here aims to investigate further these individual differences in perceptual experiences and the influence of personality, with a particular focus on the role of hemispheric asymmetries.

## Pattern recognition in vision

One could argue that the primary role of vision is to extract meaning, often from an input containing substantial amounts of uncertainty. The meaning that is imposed on that input can depend on our internal templates of prototypical stimuli (Gosselin & Schyns, 2003; Smith, Gosselin, & Schyns, 2012), our expectations and learned probabilities about the visual environment (Balcetis & Dunning, 2006; Cella, Taylor, & Reed, 2007; de Lange, Heilbron, & Kok, 2018; Grossberg, 2000; Kersten, Mamassian, & Yuille, 2004; Lau, 2008; Reed et al., 2008; Rieth et al., 2011; Schmack et al., 2013), contextual cues or prior visual input (Rensink, 2000; Yuille & Bulthoff, 1996) and on random neural fluctuations in functionally relevant areas of cortex (Hesselmann, Kell, & Kleinschmidt, 2008; Hesselmann, Sadaghiani, Friston, & Kleinschmidt, 2010; Wild & Busey, 2004). Each of these processes are also subject to influence by other factors, some of which can be attributed to the personality of the individual.

For instance, a compelling belief in the paranormal is, perhaps unsurprisingly, associated with a greater bias toward seeing faces in photographs of natural scenes (Riekki, Lindeman, Aleneff, Halme, & Nuortimo, 2012) and to imposing order in random motion and everyday events (Bressan, 2002; Riekki, Lindeman, & Raij, 2014). Believers also have a tendency to see a face in a jumbled image and to see a word in a jumble of letters (Krummenacher, Mohr, Haker, & Brugger, 2010). Overall, this observation implies a state of seeing meaning when it is not actually present in the input and that this “meaning creation” can be related to a particular belief system influencing both the expectations and templates imposed on the input.

We have recently shown that the personality dimension of Schizotypy (Claridge, 1997; Mason & Claridge, 2006; Mason, Claridge, & Jackson, 1995; Meehl, 1962), which can be closely related to paranormal beliefs (Genovese, 2005; Hergovich, Schott, & Arendasy, 2008), mediates both sensitivity and bias in the detection of faces embedded in pink (1/f) noise (Partos et al., 2016). Here we extend this work and examine whether there is any evidence for lateralization of this effect, something we might expect from the face-processing literature.

## Lateralisation of face perception

It goes without saying that the ability to detect, discriminate, and recognize a face is crucial to successful social cohesion for humans (Freiwald, Duchaine, & Yovel, 2016), and although there are well-known highly functional people who fail to recognize faces (prosopagnosia, e.g., Brad Pitt, Chuck Close, Dr Karl Kruszelnicki, “Dr P” in The Man Who Mistook His Wife For a Hat (Sacks, 1985) and Sacks himself), it can, nonetheless, be a challenging perceptual deficit.

Anatomically, there is strong evidence for face perception being lateralized to the right cerebral hemisphere (Benton & Van Allen, 1968; McCarthy, Puce, Gore, & Allison, 1997; Rhodes, Michie, Hughes, & Byatt, 2009; Rossion, Hanseeuw, & Dricot, 2012; Schiltz & Rossion, 2006; Yovel, Tambini, & Brandman, 2008; Zhang, Li, Song, & Liu, 2012). Furthermore, as is the case with many aspects of imagination, false perception, or hallucination, the brain areas responsible for the perception of illusionary faces seem to be substantially similar to those involved in actual face perception (Li et al., 2009; Rieth et al., 2011; Summerfield et al., 2005). As with actual face perception, the right fusiform gyrus consistently responds during instances of illusionary face perception (Li et al., 2009; Summerfield et al., 2005), regardless of the visual elements (such a colour, texture, shape, etc.) of the object in which the face is perceived (Taubert, Flessert, Wardle, Basile, Murphy, Murray, & Ungerleider, 2018).

Consistent with the contralateral connection between the visual hemifields and the cerebral hemispheres, the lateralisation of actual face perception to the right hemisphere produces a left visual-field (LVF) advantage for facial recognition where recognition is faster in the LVF (Hay, 1981; Hemond, Kanwisher, & Op de Beeck, 2007; Leehey, Carey, Diamond, & Cahn, 1978) and is positively correlated with the size of the right fusiform gyrus (Yovel et al., 2008). If this lateralization of face perception leads to a left visual-field advantage for facial recognition, and the same right hemisphere areas of the brain are responsible for illusory face perception, then presentation–hemifield may influence the prevalence of illusionary face perception. In one of the few studies to investigate this suggestion (Rieth et al., 2011), participants were initially trained to detect true face images embedded in noise, before being presented with noise-only stimuli (comprised of randomly distributed dark blobs) during the experimental trials. By analysing the distribution of the dark blobs in the noise stimuli, Rieth et al. (2011) found illusionary face perception was more prevalent when the dark blobs appeared to the left of the central fixation cross. These results suggest the existence of a left–hemifield bias for illusionary face perception; however, the study only presented stimuli to the central portion of participants’ visual field and did not explicitly lateralise the stimulus presentation.

Recent work has examined patterns of functional magnetic resonance imaging (fMRI)–activity during decisions about object identity and characterised two distinct processes; evidence-accumulation activity that increased as objects were revealed and recognition-activity that remained low until objects were recognized (Ploran, Nelson, Velanova, Donaldson, Petersen, & Wheeler, 2007). Subsequent work with face stimuli aligned the accumulation component with the identification of the face-likeness of an image and the recognition component with the more categorical recognition of a face and its constituent features (Meng, Cherian, Singal, & Sinha, 2012). It was further suggested that these two components of the process of facial recognition are lateralised to the left and right fusiform gyri respectively. Both these studies used the Diffusion model of decision making to characterise the accumulation of information leading to the binary decision (Ratcliff, 1978; Ratcliff & McKoon, 2008; Ratcliff, Smith, Brown, & McKoon, 2016). These studies raise a second possibility which is that if illusory face perception is more a process of evidence accumulation leading to an erroneous, false-positive response when anticipating a face, a right visual-field predisposition to see a face would be observed. This suggestion is consistent with some degree of lateralization to the left hemisphere when a face is seen purely through task-based suggestion in white noise stimuli (Smith et al., 2012).

### The current study

The study described here examines the lateralization of illusory face perception using a divided field paradigm to isolate the presentation of stimuli to the left or right visual hemifield (Bourne, 2006). The stimuli were similar to those previously used (Partos et al., 2016) with the addition of pictures of flowers, as well as faces as signal images; this was to examine whether facial recognition reflects a more general pattern recognition ability. It is not unreasonable to consider that the right hemisphere advantage for facial recognition may actually be a property of a right hemisphere advantage for generic familiar-pattern recognition (Levine, Banich, & Koch-Weser, 1988), holistic (relative) processing of disparate features (Bradshaw & Sherlock, 1982) or visuospatial task performance (Corbetta & Shulman, 2002; de Schotten et al., 2011). To examine this proposal, orchids were chosen as an alternative meaningful stimulus because their recognition requires the organization of constituent parts to form a pattern (petals, stalk, etc.), they possess similar bilateral symmetry and complexity to faces and retain the natural structure inherent in faces.

The two accounts introduced above allow us to generate two predictions about the lateralisation of illusory face/flower perception. On the one hand, if the false perception of a signal in noise is a product of specific face-processing mechanisms then we would expect to see a left-visual field advantage in the task specifically for faces and an interaction between visual field and signal-type (face/flower); on the other hand, if the percept, both real and false, is more of a process of evidence-accumulation leading to a decision about meaning in a noisy input then we would expect to see a right-visual field advantage for both faces and flowers and no interaction between visual field and signal-type.

## General methods

### Participants

Twenty-six undergraduate students (22 female), aged between 18 and 48 years (*M* = 21.54, *SD* = 6.25), completed Experiment 1 in exchange for course credit; 29 different undergraduate students (24 female), aged between 17 and 58 years (*M* = 21.31, *SD* = 8.77), completed Experiment 2. All participants had normal or corrected-to-normal vision and were righthanded (Exp 1: *M* = 9.88, *SD* = 0.43; Exp 2: *M* = 9.76*, SD* = 0.64) as assessed by the Flinders Handedness Survey (FLANDERS; (Nicholls, Thomas, Loetscher, & Grimshaw, 2013)). The study had ethical approval from the Flinders University Social and Behavioural Research Ethics Committee, and informed consent was obtained before participant involvement in the experiment.

### Apparatus

All image manipulation, coding, and presentation of experiments was carried out using Matlab (MathWorks, 2017), the GNU Image Manipulation Program and Psychophysics Toolbox (Brainard, 1997; Kleiner, Brainard, Pelli, Ingling, Murray, & Broussard, 2007; Pelli, 1997). Presentation hardware was a Dell Optiplex 3020 (with an AMD Radeon R5-240 graphics card) driving a Dell liquid-crystal display monitor (Dell, Inc., Round Rock, TX, USA) of 470 mm width and 300 mm height, at a frame rate of 60 Hz and working resolution of 1680 × 1050 pixels. The mean luminance of the display (and the stimulus images) was 45cd/m^2^ and the Commission Internationale de l'éclairage (CIE) coordinates of the white point (0.35, 0.33). The voltage to luminance relationship of the display was approximately linear over the range used through the standard gamma correction for the graphics card; this correction was considered adequate given the nature of the stimuli and the task of the observer (Partos et al., 2016); we have collected data using these stimuli on fully-calibrated Cathode Ray Tube (CRT) equipment with the same results. The numerical keypad on the righthand side of a standard USB keyboard was used to record responses. An adjustable chinrest ensured viewing distance of 570 mm in the darkened observing booth. The researcher observed participants using a closed-circuit camera to ensure ongoing attention throughout the session.

### Psychophysical stimuli

Monochrome pictures were combined with artificially generated two-dimensional noise to create visually degraded images using the novel pixel-by-pixel method of combining the signal and noise (Partos et al., 2016). This spatial degradation process meant that prespecified proportions of image pixels were randomly designated as either signal or noise pixels within a given image. Thus the degree to which the individual signal pixels correlate across space to generate a meaningful representation was degraded rather than the degree to which a single pixel in space is able to represent the signal by the signal-to-noise amplitude ratio within in the pixel. The extent to which the visual system can discriminate between individual pixels and the precise signal pixel to noise pixel structure will influence the effective difference between these two processes. We have found this method of using noise to be particularly effective in disrupting the visibility of faces and other natural objects (Partos et al., 2016), and we consider that this approach has parallels to the phase-(mis)alignment method where the continuity of edges across spatial scale is disrupted to introduce uncertainty in facial recognition (Hansen, Farivar, Thompson, & Hess, 2008; Kovesi, 2000; Morrone & Burr, 1988). Our method can also be conceptualized as a form of masking where the noise background has a percentage of pixel-sized holes that are replaced by signal (pixels), which in turn resembles the “bubbles” method used to examine the integration of spatially disparate information in tasks of recognition (Gosselin & Schyns, 2001).

**Figure 1.**
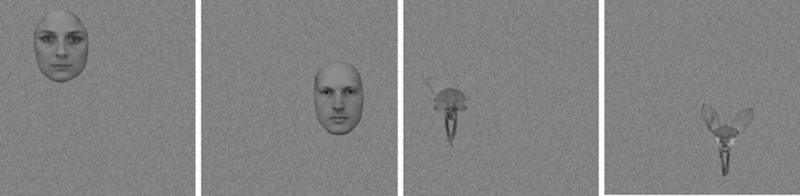
Examples of face and orchid images positioned on white noise backgrounds placed in 4 of the 8 possible positions (Left-top, right-mid/top, left-mid/bottom, right-bottom).

One male and one female facial image displaying neutral expressions in a frontal pose were sourced from the Karolinska Directed Emotional Faces Database (Goeleven, De Raedt, Leyman, & Verschuere, 2008; Lundqvist, Flykt, & Ohman, 1998). Images were cropped along the jaw and hair lines to remove all information outside of the face (see Figure 1^2^). Two images of donkey orchids (Diuris), chosen for their bilateral symmetry, were sourced online. These images were cropped to remove all information outside of the petals (see Figure 1). All images were standardized to a height of 150 pixels and converted to grayscale, and the mean luminance was matched to the average of all four (original) images. The width of the images varied slightly between the face (110 pixels) and orchid (120 pixels) categories.

**Figure 2.**
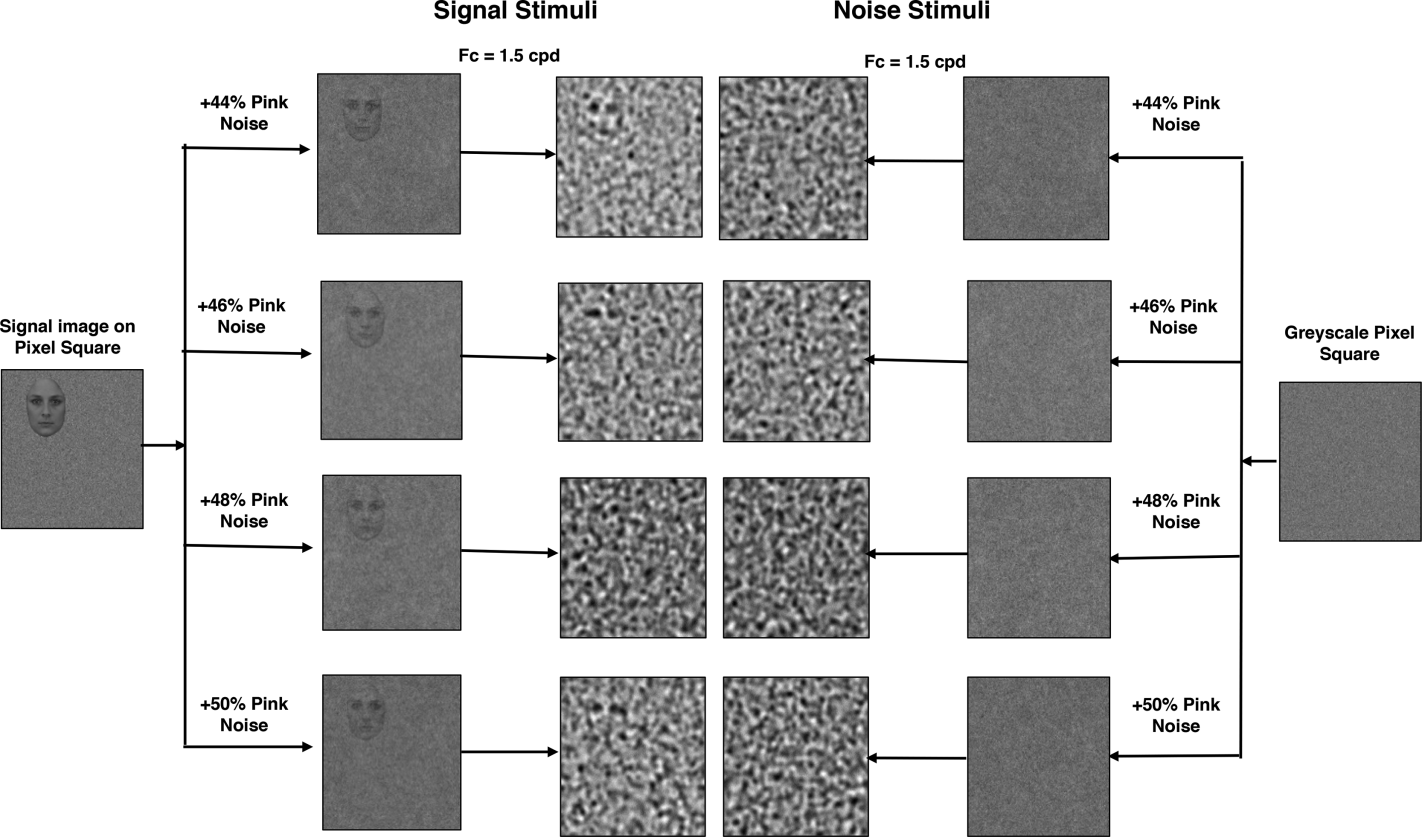
Flow-chart depicting the creation of the final stimulus set used in the experiment. F_c_ = central frequency of octave band, c/deg = cycles per degree of visual angle. In this example only one face image is shown.

To create each stimulus, the normalized eight-bit monochrome signal image was placed within a 400 × 400 pixel grid of random two-dimensional noise of similar dimensions and mean luminance and with an amplitude spectrum of 1/f (where f is the spatial frequency) to create pink-noise. The signal image was placed in eight possible locations within the grid, with four to each of the left and right sides of the center line. Within each side, the signal image could be placed at four different elevations (top, mid/top, mid/bottom, bottom). Figure 1 shows an example of each of the faces and orchids for half of the different possible signal locations. A companion 1/f noise-only image was also created with the same mean and range of pixel values and the same root mean-squared (RMS) contrast as the composite signal image. RMS contrast is generally used for nonperiodic stimuli (Frazor & Geisler, 2006; Kukkonen, Rovamo, Tiippana, & Nasanen, 1993) and for our images was in the range 0.25 to 0.35. These two images (noise-only and signal-in-noise) were then combined to create a composite image where a percentage of pixels were from the signal-in-noise image and the remainder from the noise-only image. This then creates a unique test image where a given proportion of the pixels carry the signal image embedded in the noise at a given location. To generate a stimulus set with signal images close to approximate detection threshold over the subject cohort, the composite face images comprised 16 each of 44%, 46%, 48%, and 50% noise pixels, and the composite flower images comprised 16 each of 38%, 40%, 42%, and 44% noise pixels (because they were less immediately visible during pilot testing). The image generation process meant that each image was unique and the actual visibility of a signal was dependent on that process; the rationale for choosing four levels of noise was to cover a range from almost invisible to just visible across the signal stimuli for all subjects while keeping the number of trials to a manageable level within the group design (Partos et al., 2016). A further 1/f noise-only image was also paired with each signal image as its nonsignal counterpart (also scaled in RMS to the signal image). Finally, all images were then band-pass Gaussian-filtered with an octave bandwidth at half-height centered on 1.5 cycles per degree (cpd) (6.3 cycles per face or flower image under our viewing conditions). Although this final stage of filtering may change the effective contrast of a given image, the same will be true for both signal-in-noise and noise-only images because the noise profiles are the same, and both faces and flower images are similarly scaled in size. We confirmed that the RMS contrast remained in the range 0.25 to 0.35, and as final check we visually examined all stimuli to ensure that no consistent overall contrast difference indicated the presence of a signal and thus provided a cue in the task.

As pointed out by one of our referees, a center frequency of 6.3 cycles per image is quite low for facial recognition (identity judgement), which is generally considered to be around eight to 16 cycles per image vertically, but is about right for the range of frequencies thought to signal configural face properties (detection – our current task), thought to be between two and eight cycles per image (Dakin & Watt, 2009). We also previously found a center frequency of 6.3 cycles per signal image with to be most effective at inducing false alarms for faces while retaining reasonable sensitivity to signal when it was actually present (Partos et al., 2016). Using the same stimulus profile also facilitated replication of our original experiment.

**Figure 3.**
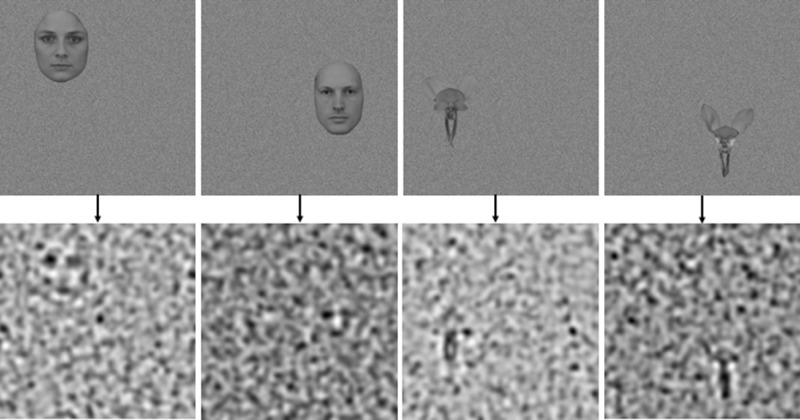
Examples of final images at 44% noise for faces, 38% noise for orchids.

In summary, a total of 128 unique images of each signal type (face/flower), distributed over four pixel-noise levels, were created along with 256 unique noise-only images. Each complete stimulus-image subtended 4.2° square of visual angle with the signal face or flower subtending 1.57° in height within that image at the 570 mm viewing distance. Stimuli were presented for 180ms in a rectangular temporal envelope. The stimulus generation process is briefly summarized in Figures 1 to 3; for further details see Partos et al. (2016).

### Questionnaires

#### FLANDERS

The FLANDERS is a 10-item self-report questionnaire assessing handedness (Nicholls et al., 2013). Scores range from −10 to +10. Only participants scoring within the right-handed range of +5 to +10 were included in this study.

#### Short Oxford-Liverpool inventory of feelings and experiences (O-LIFE)

Schizotypy was measured using the short O-LIFE (Mason, Linney, & Claridge, 2005); a 43-item, yes/no response measure. The O-LIFE has four subscales: Unusual Experiences (UnEx), Cognitive Disorganisation (CogDis), Introvertive Anhedonia (IntAnh), and Impulsive Nonconformity (ImpNon). The four subscales have concurrent validity ranging between 0.90 and 0.94 (Mason et al., 2005). Unusual Experiences questions measure positive psychotic-like symptoms, e.g., “Do you feel that your accidents are caused by mysterious forces?” Cognitive Disorganization questions measure positive disorganized symptoms, e.g., “Are you easily distracted from work by daydreams?” Introvertive Anhedonia questions measure negative symptoms, e.g., “Has dancing or the idea of it always seemed dull to you?” Impulsive Nonconformity questions measure impulsive/antisocial symptoms, e.g., “Do you often overindulge in alcohol or food?”

### Design

Experiments 1 and 2 were both presented as two blocks (face or flower) of 256 trials each. The 256 trials were presented in random order and each unique image appeared once. The order of block presentation was counterbalanced across participants. Each block was a 2 (stimuli type: signal, noise) × 2 (visual field: LVF, RVF) within-subjects factorial design with dependent variables of accuracy, response bias and response time; the continuous schizotypy measure produced dependent variable scores across the four subscales. Importantly for the measure of the false alarms, the participants were made aware of the signal-type in each block i.e., they knew what to expect and look for in the stimuli.

### Procedure

Participants were told the study was investigating differences in facial and general pattern recognition, but to avoid any effects of expectation other than those measured, the interest in visual field differences and the topic of the schizotypy questionnaire were not initially disclosed (the O-LIFE questionnaire was given after the experimental procedure). Participants were seated at the desk in the testing room and asked to complete the FLANDERS (Nicholls et al., 2013) and then adjust the chinrest to suit. The task was outlined to participants and they were asked to respond as quickly and accurately as possible using the keyboard.

The task required participants to fixate on a cross in the center of the monitor. On a single trial, a stimulus appeared for 180ms (rectangular temporal envelope) to either the left or right at 1.96° from the fixation point to the inner edge of the stimulus image. Participants were asked to report whether the image contained a signal object (face/flower) or no object. Responses were given using the “8”/up and “2”/down keys on the numerical keypad on the righthand side of the conventional keyboard. Both the meaning of these responses (stimuli present/absent), and the hand with which participants responded (left/right), were counterbalanced across participants. The next trial began 1000 ms after a response was recorded. Measures were taken of response time and accuracy. Participants were given eight practice trials during which they received feedback, before beginning the first experimental block. No feedback was given during experimental trials. Participants completed the second block immediately after the first. Upon completing both blocks, participants were asked to complete the O-LIFE questionnaire at their own pace.

### Analyses

Analyses were broadly the same for both experiments and performed using JASP (JASP_Team, 2020). We report frequentist and Bayesian statistical values here both for consistency with previous literature and to take a contemporary approach to behavioral data analysis. Bayesian statistical analysis is more tolerant to non-normal distributions of data and to smaller sample sizes and gives a more nuanced expression of any effects shown in the data. For the conventional analysis we report the p-value and an estimate of the effect size (partial Eta-squared η^2^_p_) where appropriate. For the Bayesian analysis we report Bayes Factors BF_10_, BF_M_ and BF_incl_ as appropriate. The Bayes factor, BF_10_, indicates the likelihood that the data is explained by the alternative hypothesis (compared to the null) while BF_M_ expresses the change from prior to posterior model probabilities for a given model as a result of the collected data. The former factor (BF_10_) is more commonly used but there are instances where it is meaninglessly high and it is more informative to report the BF_M_. BF_incl_ (also known as the BAWS factor) is most appropriate for the repeated measures analysis of variance (ANOVA) detailed below and is equivalent to BF_10_ for the single model conditions (JASP_Team, 2020; Jeffreys, 1961; Wagenmakers, Love, Marsman, Jamil, Ly, Verhagen, Selker, Gronau, Dropmann, Boutin, Meerhoff, Knight, van Kesteren, van Doorn, Epskamp, Matzke, de Jong, van den Bergh, Sarafoglou, Steingroever, & Derks, 2018a; Wagenmakers, Marsman, Jamil, Ly, Verhagen, Love, Selker, Gronau, Šmíra, Epskamp, Matzke, Rouder, & Morey, 2018b). We highlight any inconsistency in the reports between the two methods and leave the reader to draw their own conclusions based on both methods of analysis. The full dataset and analyses are freely available on the Open Science Framework.

#### Definitions

Throughout the article we use the term *stimulus* *type* to refer to whether the stimulus image was a signal-image (i.e., contained a face or a flower) or a noise-image (contained neither). *Signal* *type* refers to whether the signal image was a face or a flower.

#### Discrimination performance

D-prime (d’) and bias (c) were calculated to give a measure of sensitivity and bias respectively in the task (Macmillan & Creelman, 1991). The *d′* was calculated using the following:

(1) $$d^{'}=z\left(HR\right)-z\left(FAR\right),$$

where HR indicates hit rate (probability of responding “present” to signal stimuli), FAR indicates false alarm rate (probability to responding “present” to noise stimuli), and *z* indicates the transformation of HR and FAR into *z* scores. A larger *d′* indicates more accurate discrimination between signal and noise stimuli. A *d′* of 0 indicates performance at chance.

The criterion term (*c*) provides a measure of bias, or a participant's tendency to respond “present” or “not present” (Macmillan & Creelman, 1991). The *c* was calculated using the following:

(2) $$c=\;-\frac{z\left(HR\right)+z\left(FAR\right)}{2}.$$

Negative *c* values indicate a bias toward a “signal present” response, and positive *c* values indicate a bias toward a “signal absent” response. A *c* value of 0 denotes an unbiased response.

#### Reaction time

Mean reaction time was calculated by averaging reaction times for correct responses. This is inevitably a cautious measure in the current experiment given the keyboard hardware but will, on average, indicate any speed accuracy trade-offs present in the data and is suitable for the level of analysis we adopt.

#### Schizotypy

Scores for each of the four subscales of the short O-LIFE were summed. This produced individual subscale scores ranging from 0 to 12 for Unusual Experiences*,* 0 to 11 for Cognitive Disorganization, 0 to 10 for Introvertive Anhedonia, and 0 to 10 for Impulsive Nonconformity. On all O-LIFE subscales, higher scores indicate greater prevalence of relevant traits.

### Experiment 1

Experiment 1 examined the basic effect of the lateralization of seeing meaning in noisy images. Stimuli were blocked into Faces or Flowers, so the participants knew what they were looking for and were presented with a 1:1 ratio of signal-in-noise to noise-only images.

## Results

### Group sample characteristics

Assumptions of normality were verified using the Shapiro-Wilk test, kurtosis and skewness values, and visual inspection of corresponding histograms, normal Q-Q plots and box-plots. The majority of samples were normally distributed with a nonsignificant Shapiro Wilk result (*p* > .05) and kurtosis and skewness values within 2 *SD* of the mean. For the samples that produced a significant Shapiro-Wilk result and extreme kurtosis and skewness values (samples for d′ (flower stimuli to LVF), *c* (flower stimuli to LVF), and reaction time (noise stimuli in face block to LVF and to RVF)) visual inspection of corresponding figures showed the data to be suitably distributed for use of ANOVAs, which are robust against nonnormally distributed data with similar sample sizes to that of the present study (Schmider, Ziegler, Danay, Beyer, & Bühner, 2010). Assumptions of sphericity were met by design for all ANOVAs reported here because there were only two levels of any within-subjects factor.

### Sensitivity to meaning

**Figure 4.**
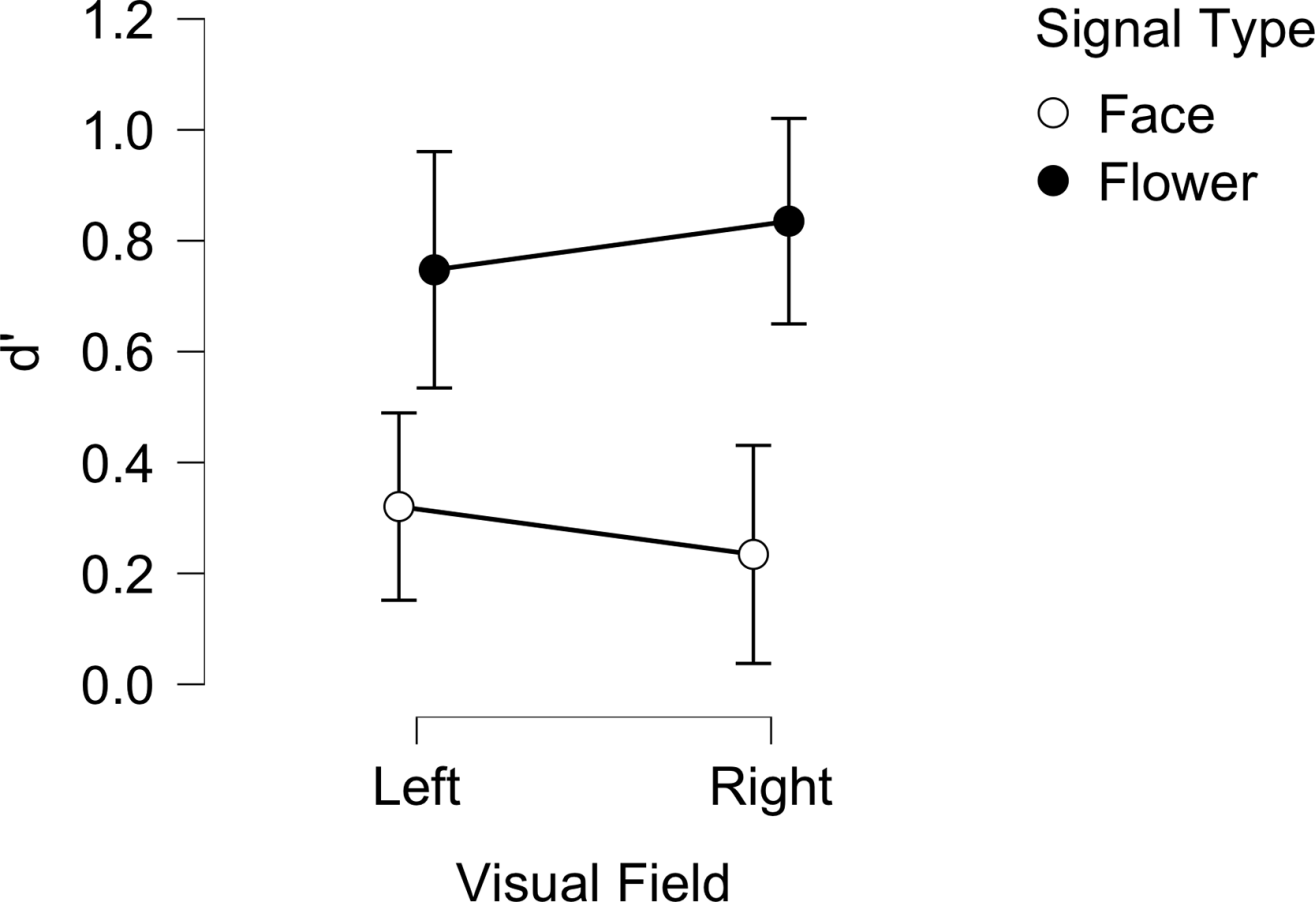
Mean *d’* for LVF and RVF across face and flower signal type blocks for the Bayesian ANOVA. Vertical lines represent ± 95% credible intervals.

Sensitivity (d′) scores were examined using a repeated measures ANOVA (and Bayesian equivalent) with the signal type (face, flower) and visual field (LVF, RVF) as within-subjects factors; the results are shown in Figure 4. In short, subjects were more sensitive to the presence of a flower than a face in both left and right visual fields, with no difference seen between the two hemifields.

Specifically, there was a significant, large main effect of signal type on d′ (*F*(1, 25) = 26.80, *p* = 2.363e-5, η^2^_p_ = 0.517; BF_10_ = 1.470e+6, BF_M_ = 13.444) which was greater for flowers (*M* = 0.791, *SD* = 0.49) than for faces (*M* = 0.277, *SD* = 0.45). There was no significant effect of visual field on the d′ value (*F*(1, 25) = 1.618e-4, *p* = 0.990, η^2^_p_ = 6.472e-6; BF_10_ = 0.202, BF_M_ = 4.236e-7) and no significant interaction between signal type and visual field (*F*(1, 25) = 1.065, *p* = 0.312, η^2^_p_ = 0.041; BF_incl_ = 0.476, BF_M_ = 0.319).

### Bias in the response

**Figure 5.**
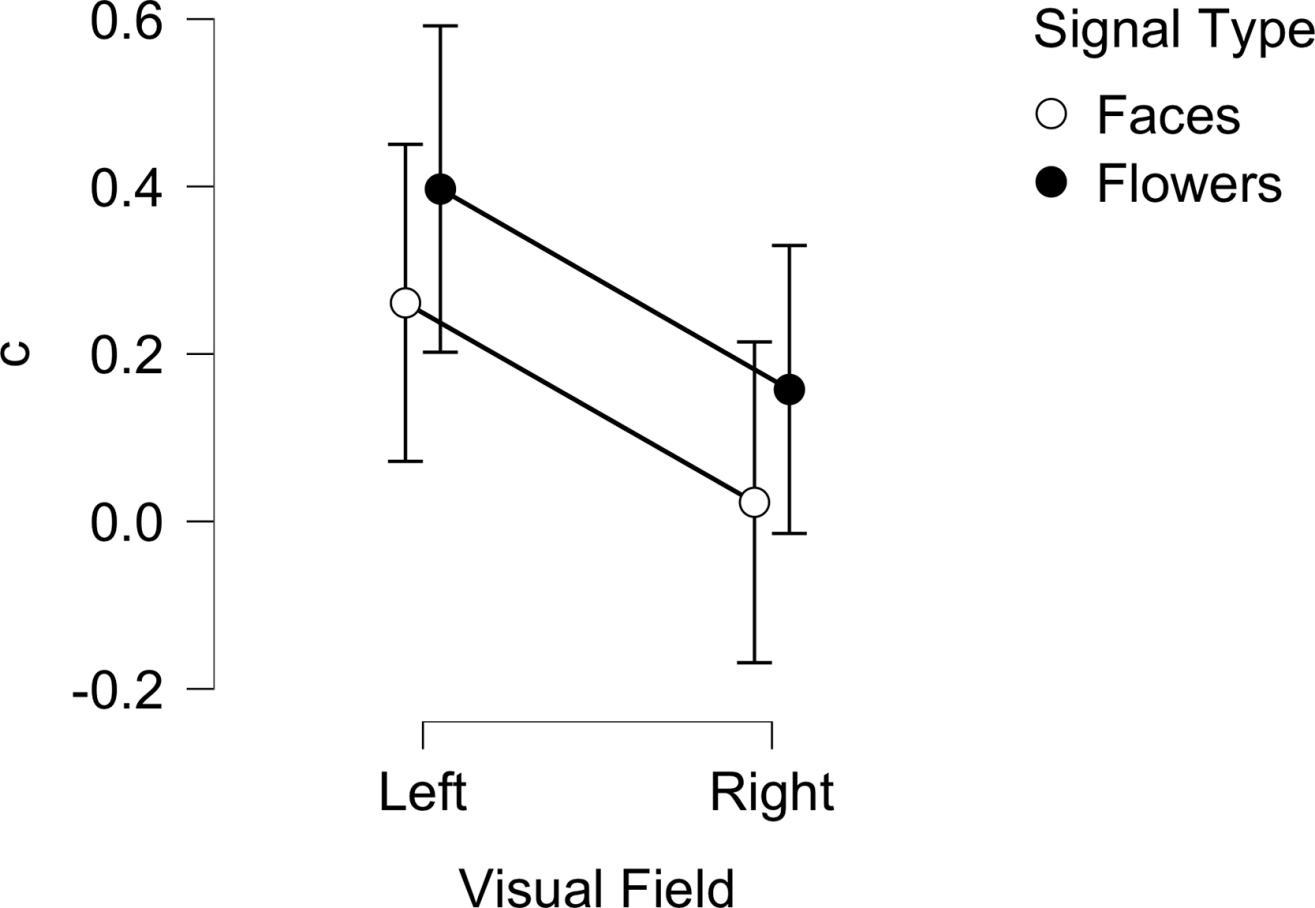
Mean *c* for LVF and RVF across face and flower signal type blocks for the Bayesian ANOVA. Vertical lines represent ± 95% credible intervals. Positive or negative scores indicate bias towards a ‘signal not present’ or ‘signal present’ response, respectively.

The bias in the response (C) was also examined through a repeated-measures ANOVA; the results for the Bayesian variant are plotted in Figure 5. These data express how a subject responds to a noise-only trial in either the “face” or the “flower” block. Overall, the expectation of a “face” elicited more false-alarms than expectation of a “flower” in both hemifields. There was also a difference between the two hemifields, with noise-only stimuli in the right visual field having a greater likelihood to elicit a false alarm (indicated by a *lower* value of c) for both faces and flowers.

Specifically, there was a significant, large main effect of visual field on the bias (*F*(1, 26) = 11.5, *p* = 0.002, η^2^_p_ = 0.315; BF_10_ = 66.07, BF_M_ = 2.078). Although both visual fields showed a bias toward a “signal not present” response (indicated by a positive value of c), this value was larger for stimuli presented to the LVF (*M* = 0.329, *SD* = 0.48) compared to the RVF (*M* = 0.09, *SD* = 0.45) indicating more false alarms in the latter. No significant effect on bias was found for signal type (*F*(1, 25) = 3.532, *p* = 0.072, η^2^_p_ = 0.124; BF_10_ = 1.147, BF_M_ = 0.024), and there was no significant interaction between signal type and visual field (*F*(1, 25) = 1.866e-4, *p* = 0.989, η^2^_p_ = 7.463e-6; BF_incl_ = 0.803, BF_M_ = 0.803).

### Reaction time

**Figure 6.**
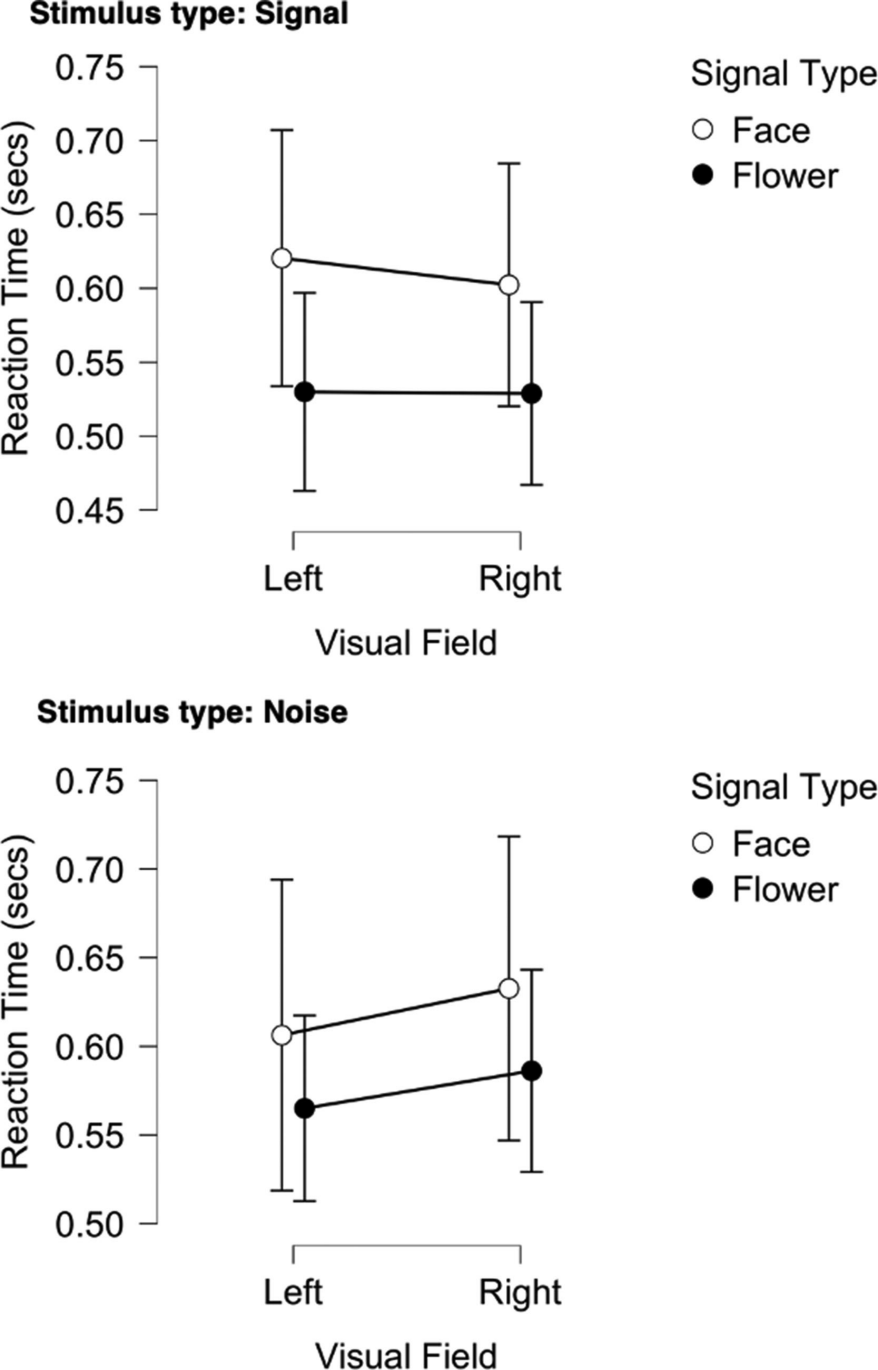
Mean Reaction time in seconds for LVF and RVF across face and flower signal (upper) and noise (lower) type blocks for the Bayesian ANOVA. Vertical lines represent ± 95% credible intervals. Note that in the noise stimulus type (lower figure) there were no signal images *per se*, just the expectation that they may be one.

The time taken to respond correctly was examined using the repeated measures ANOVA and Bayesian variant with signal type (face, flower), visual field (LVF, RVF), and stimuli type (noise, signal) as within-subjects factors (as above). Although there was no significant difference in reaction time found in the conventional ANOVA for signal type, visual field, stimulus type or for any interaction between these factors, the Bayesian alternative did suggest reasonable evidence for an effect of signal type (face, flower) (*F*(1, 25) = 3.345, *p* = 0.079, η^2^_p_ = 0.118; BF_10_ = 32.768, BF_M_ = 18.145) where it takes longer to respond to “face” in both visual fields. Examination of the mean raw reaction times for correct (hit, correct reject) and incorrect (miss, false-alarm) responses shows incorrect to have slightly longer response times than correct responses (Correct 0.584s ± 0.039; Incorrect 0.621s ± 0.055); there was no speed-accuracy trade-off in the data. Overall mean reaction times to either signal or noise images (stimulus type) are plotted in Figure 6.

### The relationship between personality and performance

To examine whether schizotypy, sensitivity, and response bias were related to one another, a Pearson *r* correlation analysis between scores on each of the four O-LIFE subscales, d′ and c for both faces and flowers was calculated. To adjust for multiple comparisons across the subscales, the critical *p*-value was Bonferroni adjusted to .0125 (0.05/4)*.* Only the “face” blocks showed any significant relationship between schizotypy and performance, i.e., when the subjects were expecting to see faces. Furthermore, only the Unusual Experiences dimension of schizotypy showed any significant correlation (shown in bold in Table 1). There was no expectation for schizotypy to be associated with visual field asymmetry for this task so the data are grouped across both right and left visual fields, but split between face and flower. These data are summarized below in Table 1.

**Table 1.** Pearson's r correlations between face/flower stimulus types and the Schizotypy subscales. Bold face indicates significant correlations (Bonferroni corrected for multiple comparisons).

	Unusual experiences	Cognitive disorganization	Introvertive anhedonia	Impulsive nonconformity
d’ face				
Pearson's r	−0.302	−0.050	−0.346	−0.189
p-value	0.133	0.807	0.084	0.354
BF_10_	0.710	0.250	1.007	0.366
c face				
Pearson's r	**−0.559**	0.101	−0.112	−0.328
p-value	**0.003**	0.622	0.585	0.102
BF_10_	**15.771**	0.273	0.281	0.866
d’ flower				
Pearson's r	−0.242	−0.368	−0.012	−0.132
p-value	0.233	0.064	0.955	0.519
BF_10_	0.478	1.233	0.244	0.296
c flower				
Pearson's r	−0.292	−0.174	−0.171	−0.187
p-value	0.148	0.396	0.404	0.361
BF_10_	0.656	0.342	0.339	0.362

**Figure 7.**
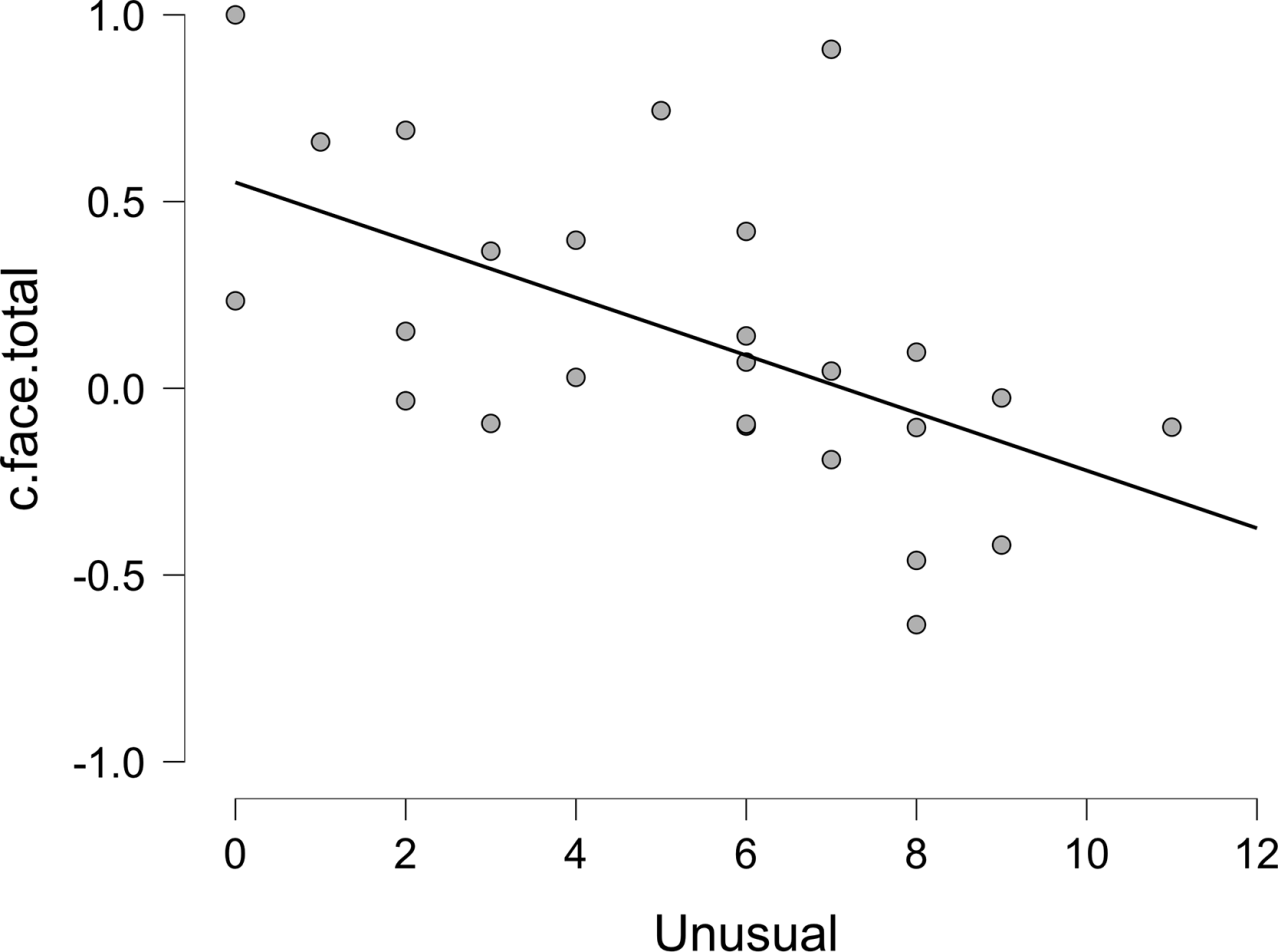
Bayesian Pearson *r* correlation between *Unusual Experiences* subscale of schizotypy and response bias for trials in the faces block collapsed across the hemifields.

The data in Table 1 suggest that the higher the Unusual Experiences (UnEx) score, the greater the likelihood of seeing a face in a noise-only. This result is represented graphically for the bias collapsed across the hemifields for the Bayesian correlation in Figure 7 and replicates the original result for this relationship between Unusual Experiences and the likelihood of seeing faces in noise (Partos et al., 2016).

**Figure 8.**
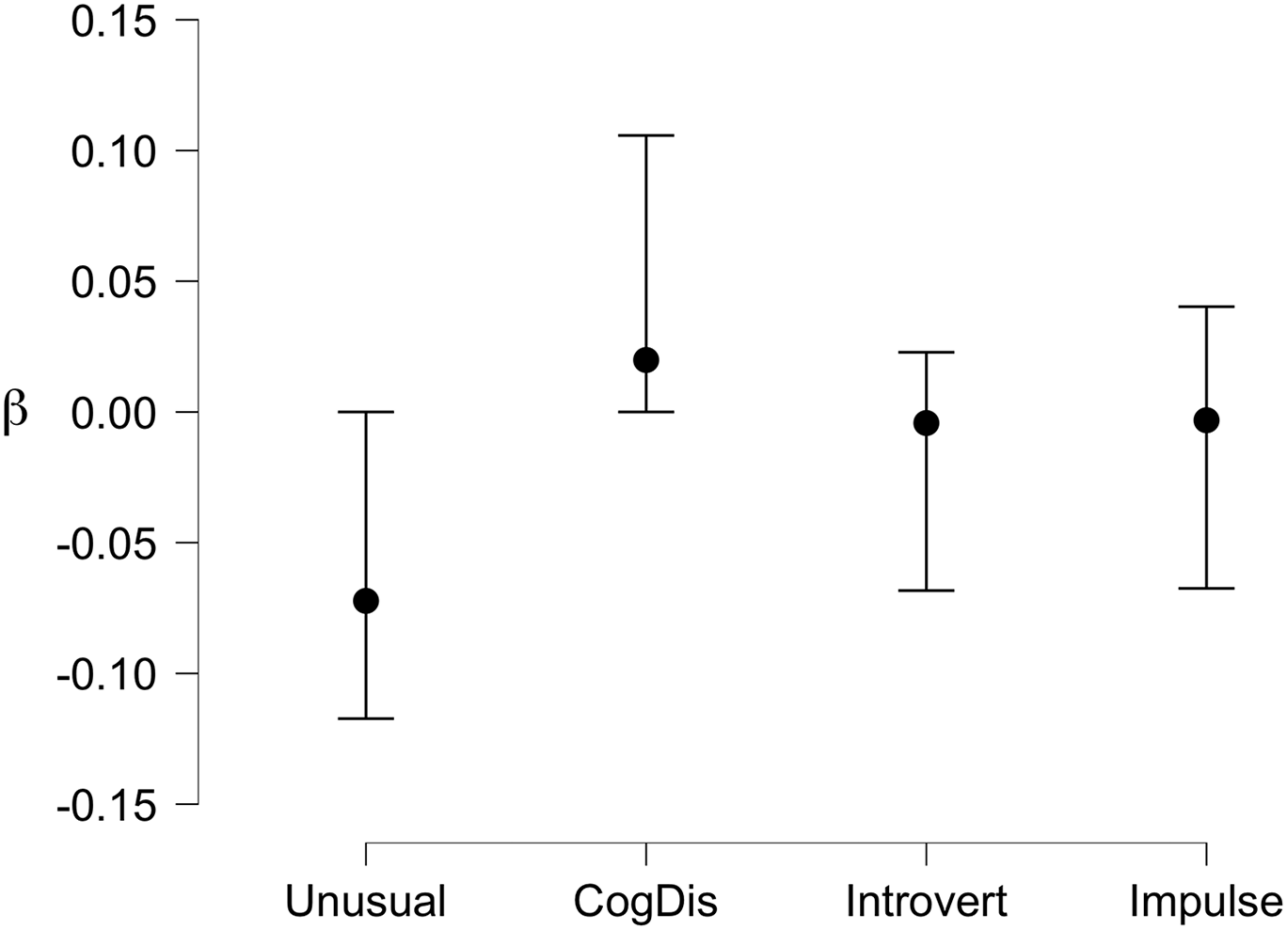
Posterior coefficients for Bayesian Linear Regression of response bias (C) for Faces against the four schizotypy subscales across both hemifields. Vertical bars are ±95% credible intervals.

To examine this effect further, linear regressions (both conventional and Bayesian) were calculated for the bias (c) for faces across both hemifields against each schizotypy subscale. We examined all data for multicollinearity; the variance inflation factor (VIF) was below 2 (max 1.484) and the tolerance above 0.6 (min 0.674) in all cases, so multicollinearity was not an issue. The full details are presented in the online dataset and model comparisons of the Bayesian regression summarized in Table 2 and Figure 8 below. The best model fit and the subscale with the largest unique influence on the data by far is Unusual Experiences (BF_10_ = 25.494, BF_M_ = 13.894) supporting the strong correlation between this personality trait and the likelihood of seeing illusory faces in noise.

**Table 2.** Model comparison (top five in order of best fit) for Bayesian Linear Regression of bias (c) for faces against the four schizotypy subscales (forced entry covariates, model comparison to null using BIC).

	Model comparison				
Models	P(M)	P(M|data)	BF_M_	BF_10_	R²
Null model	0.200	0.066	0.284	1.000	0.000
Unusual	**0.050**	**0.422**	**13.894**	**25.494**	**0.312**
Unusual + CogDis	0.033	0.178	6.281	16.118	0.372
Unusual + CogDis + Introvert	0.050	0.067	1.355	4.017	0.383
Unusual + Introvert	0.033	0.059	1.815	5.332	0.316

### Experiment 2

Experiment 2 examined the role that the actual proportion of signal images to noise images in the stimulus set might have upon the occurrence of false alarms across a trial block in a given subject. We have previously shown that the expected proportion (when the actual ratio remained at 1:1) has the predictable effect of moderating the number of false alarms in all participants (Partos et al., 2016); if they expected more signal images than were actually present, subjects made more false alarms. Here we examine the converse, whether the proportion of trials containing signal versus the proportion of trials containing only noise presented in across the stimulus block affects the results when the subjects expect the actual proportions to be equal. In this second experiment there were three times the number of noise-only trials compared to signal trials; importantly, the instructions remained identical to Experiment 1 and the subjects were expecting an equal number of noise to signal images (as in Experiment 1). All other conditions remain the same as Experiment 1.

## Results

### Group sample characteristics

Assumptions of normality were verified using the same tests as in Experiment 1: the majority of samples were normally distributed (Shapiro Wilk, *p* > .05; kurtosis and skewness values within 2 *SD* from the mean). The samples for *c* (face stimuli to LVF; flower stimuli to RVF) and reaction time (noise stimuli in face block to LVF and to RVF) produced a significant Shapiro-Wilk result and extreme Kurtosis and Skewness values although, as in Experiment 1, inspection of corresponding figures showed the data to be suitably distributed for use of ANOVAs (Schmider et al., 2010).

### Sensitivity to meaning

**Figure 9.**
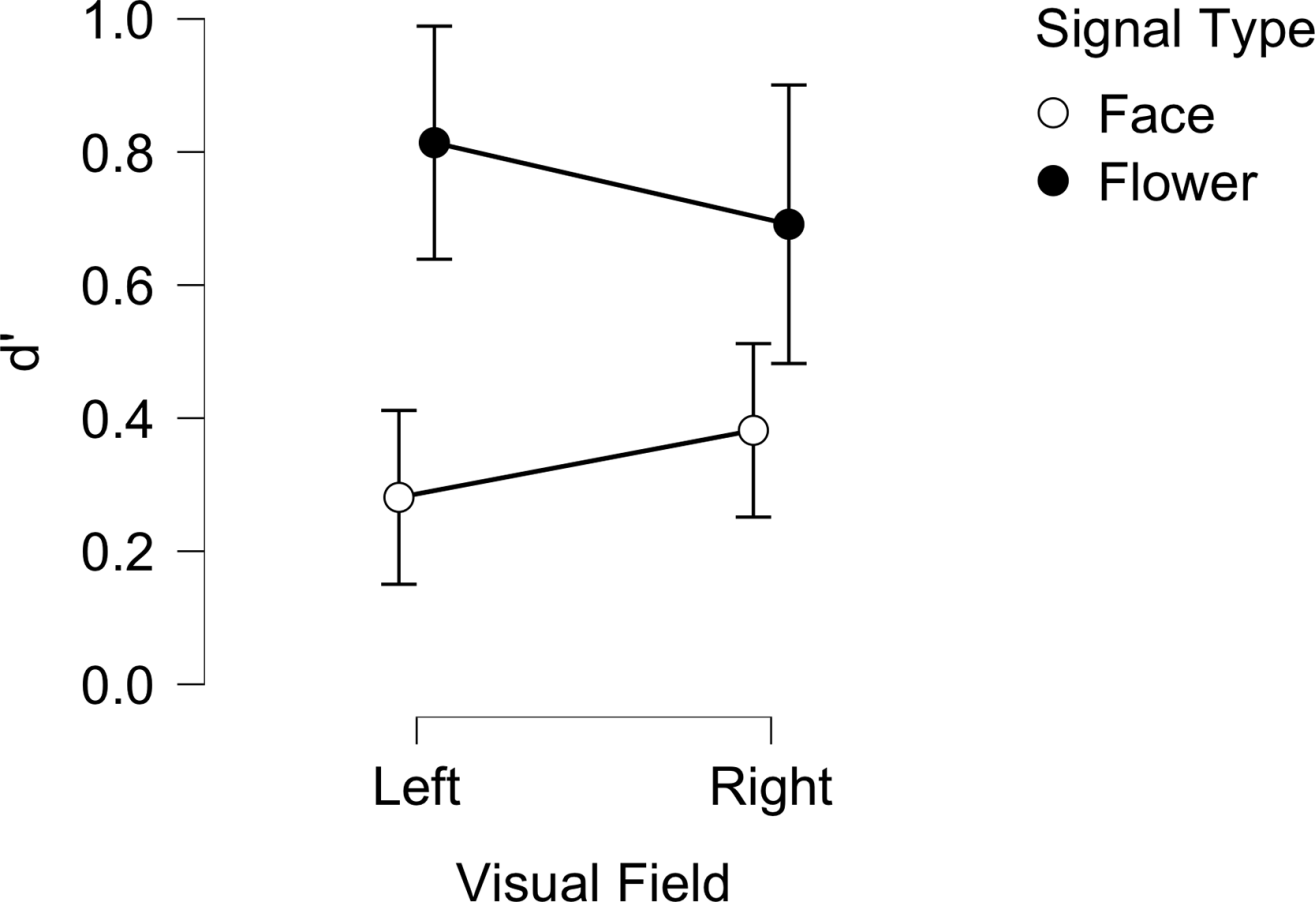
Mean *d’* for LVF and RVF across face and flower signal type blocks for the Bayesian ANOVA. Vertical lines represent ± 95% credible intervals.

Sensitivity to the presence of a signal image (d′) was analyzed using the same ANOVA approach as in Experiment 1. The results are broadly the same as Experiment 1 with sensitivity being greater for flowers than faces in both hemifields (Figure 9).

Specifically, there was a significant main effect of signal type on discrimination (*F*(1, 28) = 33.387, *p* = 3.330e-6, η^2^_p_ = 0.544; BF_10_ = 1.056e+6, BF_M_ = 10.209), and sensitivity was significantly greater for flowers (*M* = 0.75, *SD* = 0.51) than for faces (*M* = 0.33, *SD* = 0.34). There was no significant effect of visual field on discrimination (*F*(1, 28) = 0.025, *p* = 0.875, η^2^_p_ = 8.944e-4; BF_10_ = 0.197, BF_M_ = 1.349e-7) and no significant interaction between signal type and visual field (*F*(1, 28) = 3.67, *p* = 0.07, η^2^_p_ = 0.116; BF_incl_ = 0.633, BF_M_ = 0.633).^1^

### Bias in the response

**Figure 10.**
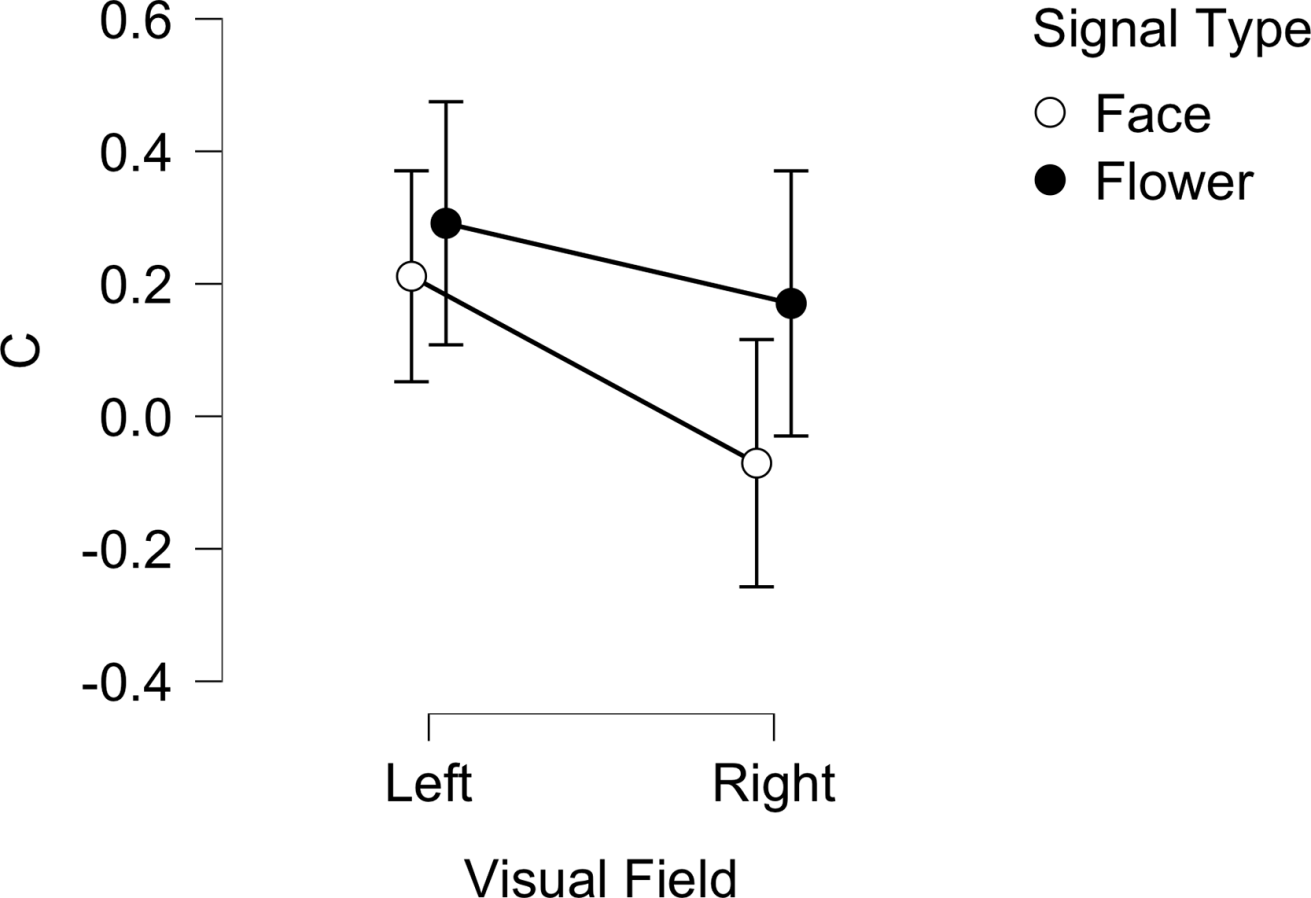
Mean *c* for LVF and RVF across face and flower signal type blocks for the Bayesian ANOVA. Vertical lines represent ± 95% credible intervals. Positive or negative scores indicate bias towards a ‘not present’ or ‘present’ response, respectively.

The response bias (c) was examined similarly to the sensitivity; the results are plotted in Figure 10. Again, the data largely mirror Experiment 1, although there is a stronger effect for faces in the right visual field. This indicates that individuals were more likely to see a face in a noise-only image in the right visual field, and this effect was exacerbated specifically for this stimulus-type, i.e., a noise-only image when the subject was looking for faces.

There was a significant main effect of visual field on bias (*F*(1, 28) = 9.876, *p* = .004, η^2^_p_ = 0.261; BF_10_ = 4.486, BF_M_ = 1.388). Both visual fields showed a bias toward a “not present” response for both stimulus types when combined, but this bias was greater for stimuli presented to the LVF (*M* = 0.23, *SD* = 0.45) compared to the RVF (*M* = 0.07, *SD* = 0.51); the RVF showed a bias toward saying a face was “present” in the face stimulus-block, indicated by a c value less than 0. No significant effect on bias was found for signal type *(F*(1, 28) = 2.999, *p* = 0.097, η^2^_p_ = 0.261; BF_10_ = 1.376, BF_M_ = 0.343), and there was no significant interaction between signal type and visual field (*F*(1, 28) = 1.70, *p* = 0.203, η^2^_p_ = 0.057; BF_incl_ = 0.425, BF_M_ = 0.882).

### Reaction time

**Figure 11.**
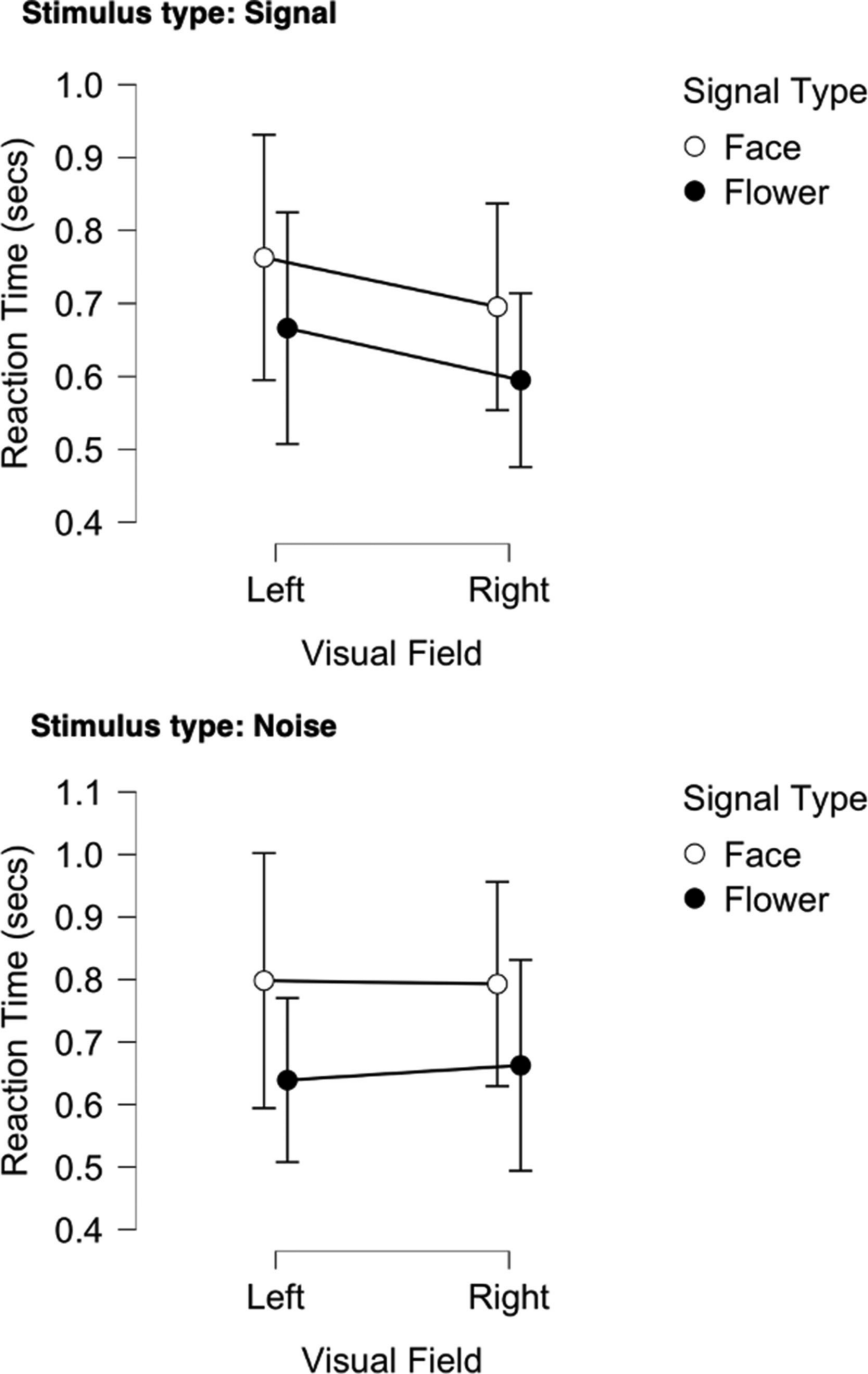
Mean Reaction time in seconds for LVF and RVF across face and flower signal (upper) and noise (lower) type blocks for the Bayesian ANOVA. Vertical lines represent ± 95% credible intervals. Note that in the noise stimulus type (lower figure) there were no signal images *per se*, just the expectation that they may be one.

**Figure 12.**
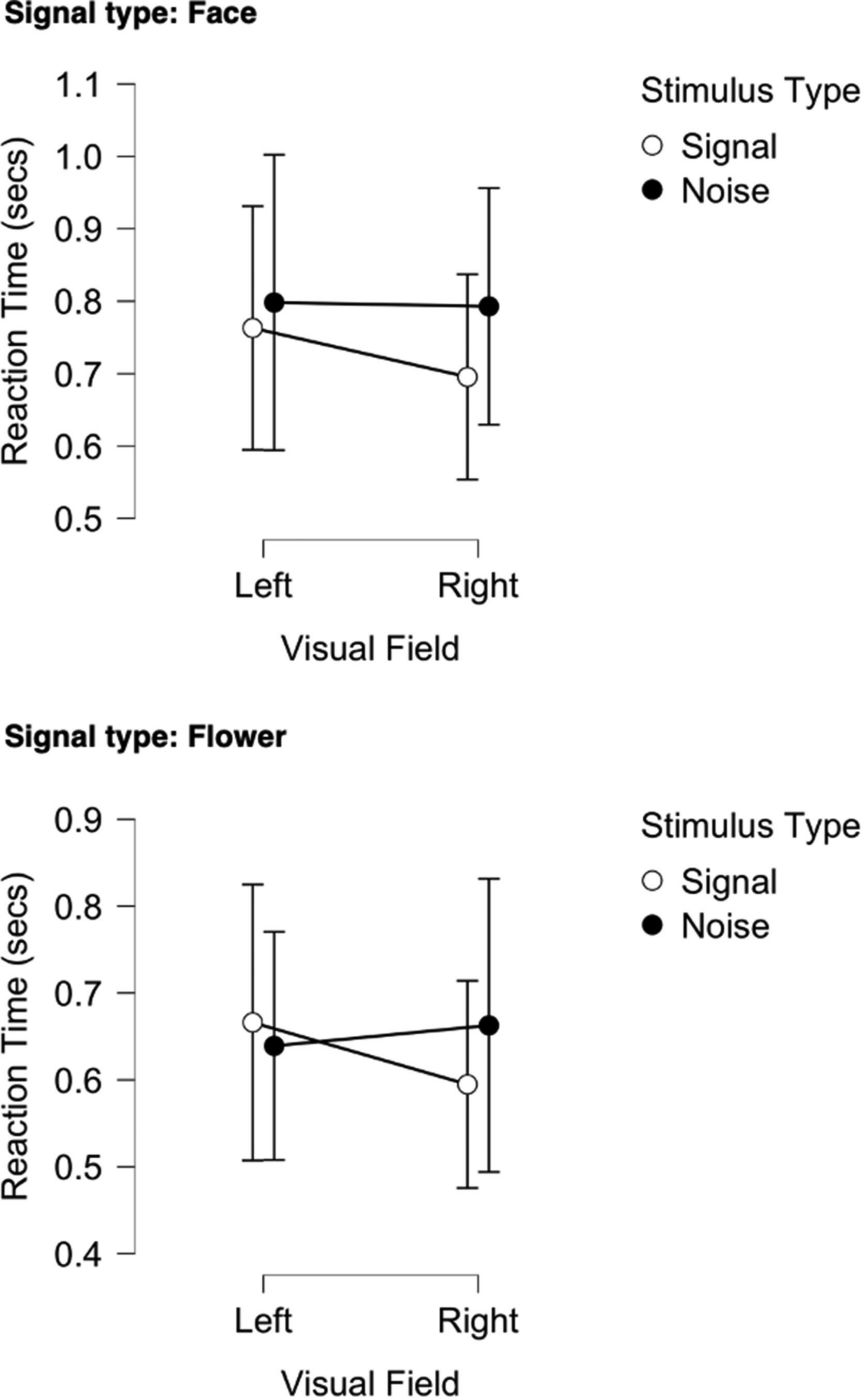
Mean Reaction time in seconds for LVF and RVF across signal and noise trials for face (upper) and flower (lower) type trials for the Bayesian ANOVA. Vertical lines represent ± 95% credible intervals.

Reaction time was analyzed using the same ANOVA approach as in Experiment 1 with the results shown in Figure 11. As in Experiment 1, there was no evidence for any speed-accuracy trade-off in the data (Mean reaction times: Correct 0.702s ± 0.075; Incorrect 0.73s ± 0.07). There was a significant effect of response time in that correct identification of faces being present was significantly slower than flowers in both hemifields (*F*(1, 28) = 4.770, *p* = 0.037, η^2^_p_ = 0.146; BF_10_ = 305.819, BF_M_ = 18.319). The interaction between visual field and stimulus type (signal, noise) was just significant (*F*(1, 28) = 4.939, *p* = 0.035, η^2^_p_ = 0.150; BF_incl_ = 0.403, BF_M_ = 0.356) and suggestive of a faster response to a signal stimulus in the right visual field compared to the left for faces and flowers (see Figure 12). Overall, mean reaction times were faster in Experiment 1 than Experiment 2 by around 100 ms (grouped marginal means from Bayesian ANOVA: Exp 1 = 0.584 ± 0.04, Exp 2 = 0.702 ± 0.075; T-test = 0.002, F-test = 0.109).

### The relationship between personality and performance

**Figure 13.**
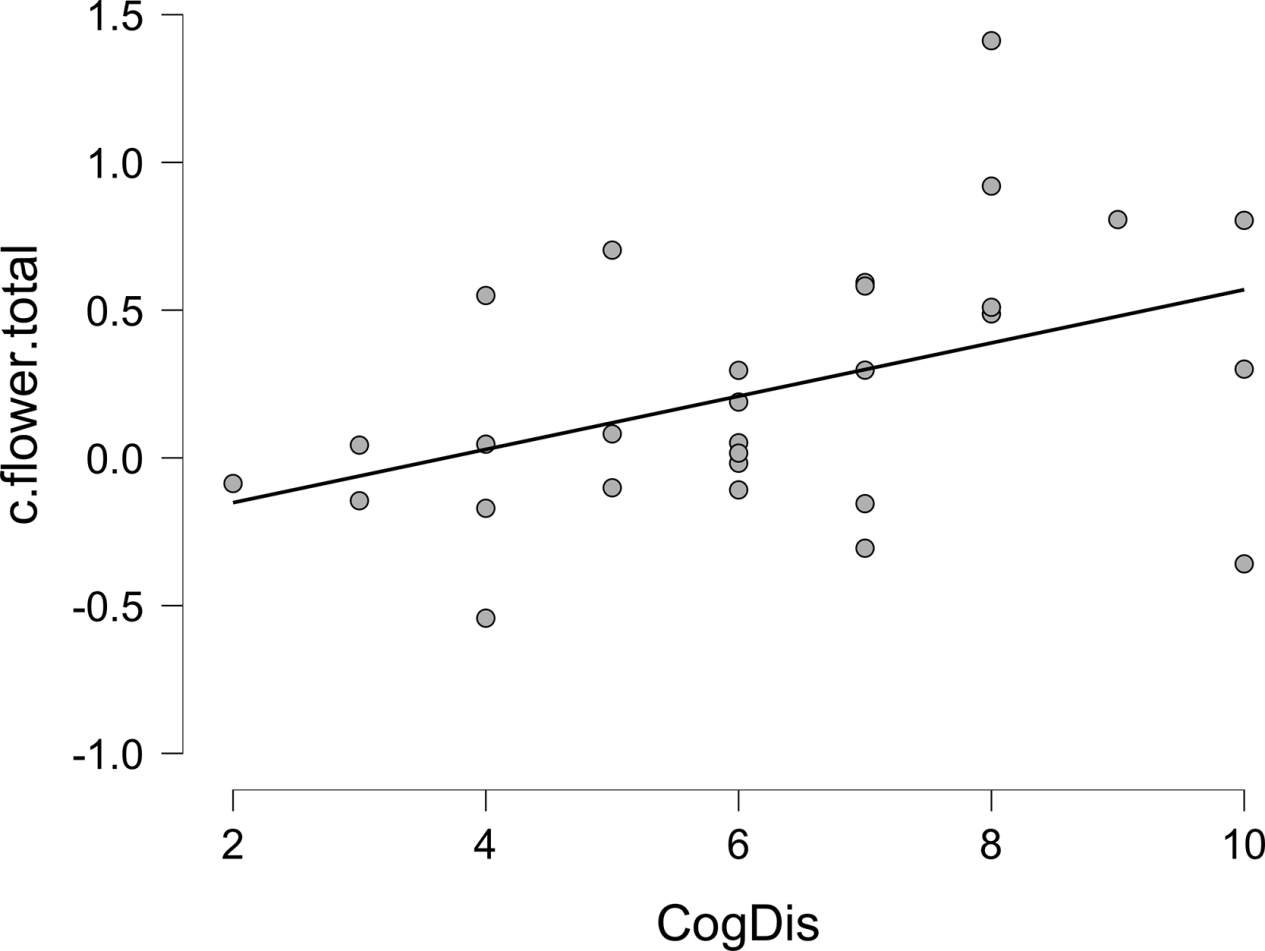
Bayesian Pearson *r* correlation between *Cognitive* *Disorganization* (CogDis) subscale of schizotypy and response bias for trials in the flowers block across both hemifields.

As above, a Pearson's correlation between the scores on each of the four O-LIFE subscales, d′ and *C* for both faces and flowers was calculated to examine the relationship between personality and performance on the task. The only significant correlation in this data was a *positive* relationship between the response bias for the flower stimuli and the Cognitive Disorganization subscale, indicating that the higher the score, the less likely the individual was to indicate a “flower” present in a noise stimulus or the more conservative their response. When Bonferroni-corrected for multiple comparisons, however, this relationship did not reach significance in terms of the *p* value, although the Bayes factor (BF_10 =_ 3.344) suggested moderate evidence of the relationship. Table 3 shows the results of the correlations in brief, the full data set is available online, and Figure 13 plots the correlation for the Bayesian variant.

**Table 3.** Pearson's r correlations between face/flower stimulus types and the Schizotypy subscales. Bold face indicates significant (uncorrected) correlations.

	Unusual experiences	Cognitive disorganization	Introvertive anhedonia	Impulsive nonconformity
d’ face				
Pearson's r	−0.030	0.022	−0.081	0.238
p-value	0.876	0.911	0.678	0.214
BF_10_	0.233	0.232	0.499	0.482
c face				
Pearson's r	−0.314	0.181	−0.114	−0.053
p-value	0.097	0.346	0.557	0.785
BF_10_	0.859	0.352	0.231	0.239
d’ flower				
Pearson's r	0.040	−0.164	−0.039	−0.049
p-value	0.835	0.396	0.842	0.801
BF_10_	0.236	0.325	0.171	0.238
c flower				
Pearson's r	0.121	**0.437**	−0.012	0.135
p-value	0.532	**0.018**	0.952	0.484
BF_10_	0.278	**3.344**	0.195	0.291

**Figure 14.**
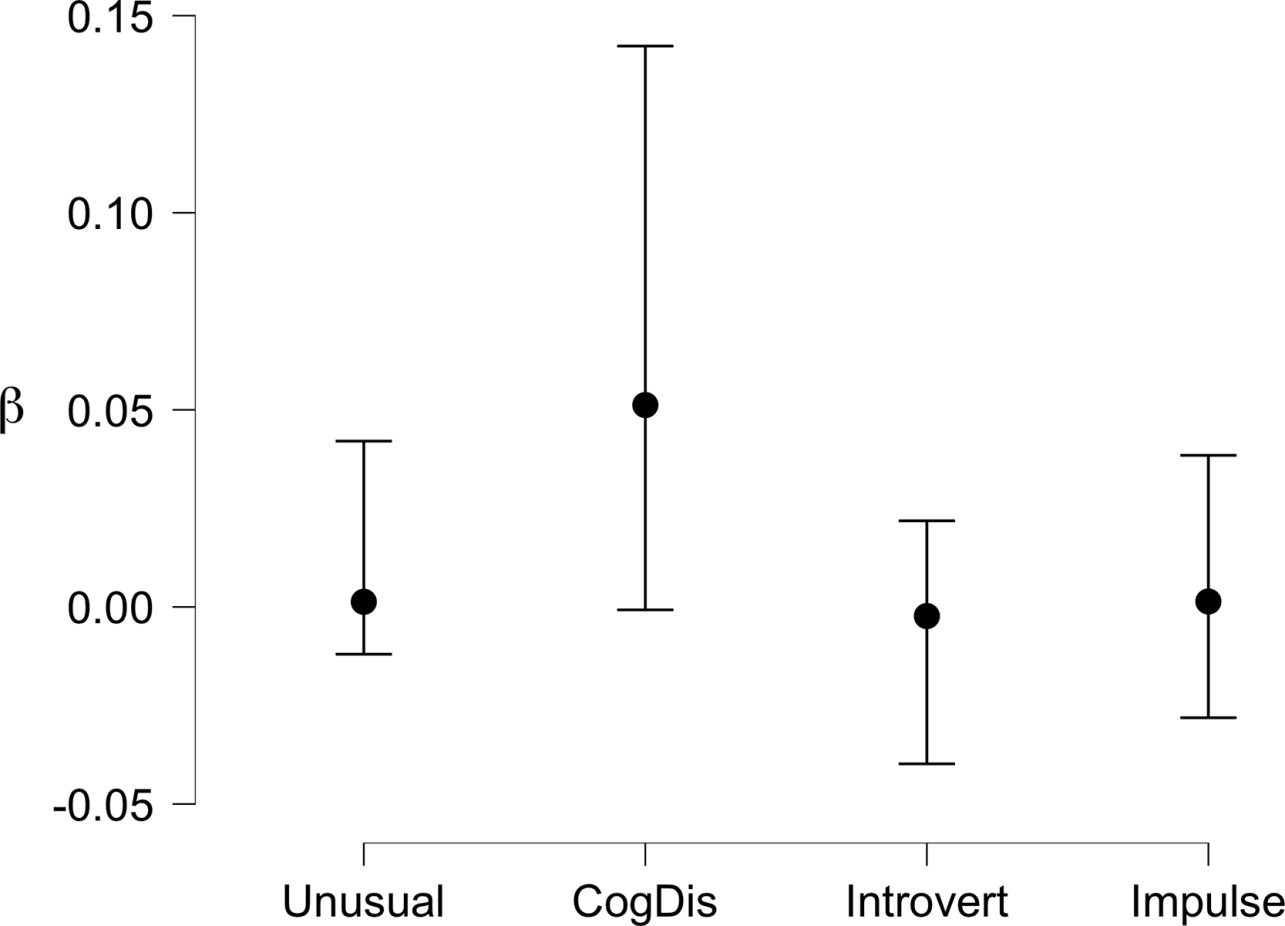
Posterior coefficients for Bayesian Linear Regression of response bias (C) for Flowers against the four schizotypy subscales across both hemifields. Vertical bars are ±95% credible intervals.

A linear regression (conventional and Bayesian) was calculated for the bias (c) for both face and flower stimuli against the schizotypy subscales. Multicollinearity was not an issue with the VIF below 2 (max = 1.270) and the tolerance above 0.6 (min = 0.788). Table 4 shows the top five models for the Bayesian regression, and Figure 14 plots the posterior coefficients and credible intervals. The data is best described uniquely by the Cognitive Disorganization subscale, supporting the moderate relationship brought out by the correlation.

**Table 4.** Model comparison (top five in order of best fit) for Bayesian Linear Regression of bias (C) for Flowers against the four schizotypy subscales (forced entry covariates, model comparison to null using BIC).

	Model comparison				
Models	P(M)	P(M|data)	BF_M_	BF_10_	R²
Null model	0.200	0.364	2.292	1.000	0.000
CogDis	**0.050**	**0.364**	**10.864**	**3.995**	**0.191**
CogDis + Introvert	0.033	0.054	1.652	0.888	0.201
Unusual + CogDis	0.033	0.046	1.413	0.765	0.192
CogDis + Impulse	0.033	0.045	1.372	0.744	0.191

## Discussion

The experiments presented here examined the effect of signal location in either visual hemifield on the ability to detect either a face or a flower in noise and the role that personality has upon the task through the lens of the schizotypy subscales. We presented equal proportions of signal and noise images in a trial-block in the first experiment and three-times as many noise-only images in in the second experiment. Across both experiments, sensitivity (d′) was greater for flowers than faces, but there was no lateralization for either signal-type. In Experiment 1 there was a visual-field asymmetry seen in the likelihood to see a signal when one was not present (c). This predisposition to visual false alarms was biased toward the right visual field more than the left for both faces and flowers and positively correlated to the Unusual Experiences schizotypy subscale (Partos et al., 2016). Experiment 2 showed a similar right visual field bias to false alarms, with a stronger bias toward saying “face’ when none was present for all subjects, but this did not correlate to the Unusual Experiences subscale; there was a moderate positive relationship between the Cognitive Disorganization subscale and the bias score when the subject was expecting a flower (indicating a lower likelihood of saying “flower” in a noise-only image). Finally, although there was no asymmetry in sensitivity (d′) there was a moderate suggestion of a faster response to a signal (both face and flower) in the right visual field in Experiment 2. In terms of the predictions made in the introduction these data support an evidence-accumulation explanation of seeing meaning in a noisy input, particularly when seeing something when it is not actually there.

### Accumulation of evidence or recognition of an object?

The lack of any apparent advantage in the task for stimuli presented in the left visual hemifield is, on the face of it, inconsistent with the literature on face detection and identification but fits with the recent observations on object/face identification and evidence accumulation (Meng et al., 2012; Ploran et al., 2007). When one considers the task at hand, this makes some sense. The decision made by the subject was whether there was a stimulus present in the noise; they knew whether that stimulus was meant to be a face or a flower on a given trial and the task was a yes/no signal-present task. This decision is a necessary, but not sufficient, process for the recognition of a face or a flower (or any object), which tends to have been the more demanding task that the majority of the literature has been concerned with. One of the novel aspects of this study is that the stimuli are specifically constructed to maximize the likelihood of seeing something when it is not necessarily there, akin to seeing faces in the clouds or hearing your name spoken in a crowded room full of voices (Partos et al., 2016). The spatial structure of the noise mimics the statistics of natural scenes and, arguably, the operation of natural systems such as the brain. The signal is embedded in the noise on a pixel-by-pixel basis to create meaning that evolves through correlation across space as the signal-pixel–to–noise-pixel ratio increases. The task of the subject is to decide if a signal is present in the noise as quickly and accurately as possible. This process of making a binary decision in a noisy stimulus has been formalized in several ways, but one model that has gained traction as an explanatory framework for much of the data is the Drift Diffusion Model of binary decisions (Ratcliff, 1978; Ratcliff & McKoon, 2008; Ratcliff et al., 2016). The success of the Diffusion model lies in its transparent simplicity and that has been effective in linking both behavioral and neurophysiological data within the same framework (reviewed in Ratcliff et al. (2016)). Given its use in explaining the imaging data corresponding to object and facial recognition (Meng et al., 2012; Ploran et al., 2007) we discuss its potential in explaining our data here, but recognize that other models of signal detection in noise (e.g., the Linear Amplifier model and its variants, or the more similar Linear Ballistic Accumulator model) may work equally well (Donkin, Brown, Heathcote, & Wagenmakers, 2011; Lu & Dosher, 2008).

The Diffusion Decision Model is a process whereby one of two alternative outcomes is chosen after a period of time of indecision, during which the (simulated) neural activity drifts toward one or other of the opposed alternatives as evidence is accumulated. This change in the activity (nominally in polarity around a resting potential of zero) has a rate of change over time (the ‘drift rate’) and a decision may be made when the activity reaches a particular threshold level. This level is generally balanced in magnitude either side (+/-) of zero. The time taken to reach the decision can be affected by both the rate and the threshold and careful titration of the stimulus properties and the task in relation to the decision allows significant insight into the underlying process. The particular stimulus structure used here, the relatively short presentation time for the task and the speeded response makes the data ideal for explanation in terms of the Diffusion model.

While it is notoriously hard to correlate the behavior of any computational model to neural activity, the Diffusion model is arguably a reasonable representation of the way in which neurons change their membrane potential until a point at which an action potential is initiated, constituting the “decision” to pass that signal on. Two neurons with equal and opposite sensitivity could constitute the primary components of a simple diffusion process. The model is more commonly related to a more global measure of activity in certain regions of the brain; it is this second form of implementation that provides a possible explanation for our data within the context of facial and object recognition measured with fMRI (Meng et al., 2012; Ploran et al., 2007).

It is equally hard to equate imaging data to underlying neural activity on anything but a gross scale. However, when subjects are shown gradually appearing images and asked to respond as soon as they recognize the image, fMRI of bilateral occipital brain areas (including the fusiform gyri) shows a change in blood flow consistent with accumulation of information to a point at which a decision can be made (Ploran et al., 2007). Other areas showed evidence for a more sudden peak activity at the point of image recognition (Ploran et al., 2007), suggesting a separation between and functional localization of discrimination (signal from noise) and recognition. Although the images used in these experiments were not confined to faces, later imaging studies showed a distinction between left and right fusiform gyri in their accumulated activity to stimuli varying in their resemblance to a face (Meng et al., 2012). Activity in the left fusiform gyrus corresponded with the degree to which a given stimulus resembled a face, whereas activity in the right fusiform gyrus peaked at the point at which a “face” decision was made and endured after the left fusiform activity had abated (Meng et al., 2012). This second result is consistent with the topography of the EEG activity when subjects saw a face in white noise through pure suggestion; no face ever being present (Smith et al., 2012). Overall these results suggest a lower-level evidence-accumulation role of the left fusiform before face categorization and recognition by the right fusiform gyrus (McCarthy et al., 1997; Rhodes et al., 2009; Yovel et al., 2008; Zhang et al., 2012), and we suggest an evidence-accumulation process of the kind described by the Diffusion model lateralized to the left hemisphere mediates the illusory percept of faces and flowers seen in our stimuli. The lack of any left-visual field advantage in the results or of any interaction between visual-field and signal-type suggests that the deeper facial recognition process mediated by the right fusiform gyrus, apparent in much of the face processing literature, is not fully engaged in our task and having a negligible effect on our data. The increased incidence of false-alarms in the right visual field, particularly for faces but also for flowers, suggests this illusory image response may be an error of evidence-accumulation leading to a false alarm rather than an error of signal recognition per se: an observation we consider to be consistent with the stimulus and task properties.

### Personality, laterality and false-alarms

The framework provided by Diffusion model to contextualize the data presented here might also make sense of the personality data and the increased likelihood to respond “face” to a noise-only stimulus, i.e., make a visual false alarm, which is affected by both personality and visual field. We have previously shown that the Unusual Experiences dimension of the schizotypy subscales is positively correlated to the likelihood to make a visual false alarm (and therefore negatively correlated with c) and to see a face in noise when one is not actually present. This result is replicated in Experiment 1 and suggested in the data of Experiment 2; the current results also show a greater bias toward making a false-positive result in the right visual field for both faces and flowers. This lateralization of the original result (Partos et al., 2016) is consistent with the suggestion that the majority of the measured response in the current data is mediated by the left fusiform gyrus (Meng et al., 2012; Ploran et al., 2007); both correct and incorrect responses being mediated by the same underlying mechanism, it is just the ultimate decision made that differs. Wheras both faces and flowers showed similar bias, the expectation of a face was slightly more likely to elicit a false alarm; this is again consistent with the original result and also the observations concerning the lateralized activity of the fusiform gyri particularly in response to face stimuli (McCarthy et al., 1997; Rhodes et al., 2009; Yovel et al., 2008; Zhang et al., 2012). We, and others, have suggested that a higher score in positive schizotypy elicits perceptual properties consistent with a noisier system (Badcock, Waters, Maybery, & Michie, 2005; Koychev, Deakin, Haenschel, & El-Deredy, 2011; Partos et al., 2016; Winterer, Coppola, Goldberg, Egan, Jones, Sanchez, & Weinberger, 2004) and the effect that the internal noise has on perceptual decision-making (Pelli, 1981). Given our explanation above in terms of evidence accumulation and the Diffusion model, we would expect this role of personality to follow the same pattern of lateralization as seen more generally leading to more illusory signals in the right visual field when that predisposition is present in the individual.

### Discrepancies

There are some discrepancies in the current work both with the larger body or literature on face recognition, between Experiments 1 and 2, and with our previous work. These differences, we suggest, are founded in both the proposed underlying mechanisms at play and the precise experimental conditions used in the collection of each data set.

The most interesting discrepancy, that with the larger body of literature on face perception, is largely explained by the explanation for our data offered above regarding the task at hand and the particular stimulus properties. We would expect the more commonly observed left-visual field advantage to return if the task were facial recognition. To examine this suggestion, we also conducted a study where the task was “face or flower” and the stimulus always contained an image (either face or flower) and so an object recognition process must be engaged to complete the task. We found no lateralizing effect of visual field on either d′ or c (data available on request). There was, however, no explicit return of the proposed left visual field advantage in recognition which could be a product of the stimulus structure and the task: generally speaking the left-visual field advantage is found for more traditional “face” stimuli and recognition of specific facial properties.

The only difference between Experiments 1 and 2 was in the signal-in-noise to noise-only image ratio (1:1 compared to 1:3, respectively); although the subjects were not told of this change they would clearly build up a frame work of the stimulus-presentation statistics through recent experience. A likely outcome of this mismatch between expectations and evidence is an increase in uncertainty in the response, which is consistent with the overall longer reaction times in Experiment 2. These expectations, explicit and implicit, have been shown to affect what we see (Cella et al., 2007; Partos et al., 2016; Smith et al., 2012). The discrepancy in the results between Experiments 1 and 2 was principally in the lack of any correlation of bias (c) with Unusual Experiences (although c remained lateralized to the right visual field) and a negative correlation between Cognitive Disorganization and likelihood to see a flower in a noise-only image (a positive correlation with c).

It is worth noting that the directions of most correlations were the same between Experiments 1 and 2 and, furthermore, they were largely also consistent with the original study for faces (Partos et al., 2016). The reduced effect of Unusual Experiences in Experiment 2 is actually consistent with the observation that subjects higher in this subscale score were less likely to make false alarms to word stimuli when presented with fewer signal stimuli than expected (Cella et al., 2007). This interaction may have had a moderating effect on the relationship between the false alarms to faces and Unusual Experiences. Combined with the result that overall all subjects made more false-alarms to faces in the right visual field in Experiment 2 makes this lack of interaction less concerning.

The positive correlation of the bias score (c) with Cognitive Disorganization in Experiment 2 was moderate (BF_10_ = 3.344) and indicated that the higher the score in this subscale, the more conservative the responses in the flower blocks (see also Partos et al. (2016), Experiment 1). We originally suggested in Partos et al. (2016) that this result might be an effect of increased social anxiety and neuroticism (which correlates positively with Cognitive Disorganization (Claridge et al., 1996)) and subjects being more conservative in their responses, particularly when there appear to be fewer signal images than expected, as is the case in the current Experiment 2. The increased visibility of the flower stimuli relative to the face stimuli (shown by the higher sensitivity scores), possibly because of the elongated petal forming a visible streak in the image acting as a cue, may explain why this relationship was principally confined to flowers. Subjects were possibly more aware of the greater number of noise stimuli in a flower block which then moderated their responses accordingly. Although every effort was made to make the all the signal-images equally visible for given percentage of noise-pixels for both faces and flowers, the image generation process makes this close to impossible even through pilot testing because each image is spatially unique. We feel that this spatial property of the stimuli is an important factor in the use of these stimuli and potentially mirrors the internal representation of any noisy stimulus; the basis on which the system must decide whether a signal is present or not.

## Conclusions

The data presented here suggest that the left visual field advantage previously shown for tasks of facial recognition is diminished and, in some cases, reversed when the task is a simpler signal-detection task. We have contextualized the data in terms of the task at hand, the complementary roles of the left and right hemispheres in the process of image detection, discrimination and recognition, and the rapid evidence accumulation process well described by a process along the lines of the Diffusion Decision model.

## Footnotes

1 Strictly speaking, and as pointed out by one of our reviewers, these values should be d′a and ca due to the (likely) unequal variances of signal and noise stimuli in Experiment 2. However, the conclusions for the statistical analysis will remain the same because the numbers will be in the same proportions. For consistency with Experiment 1 we chose to keep d′ and c as the raw calculations.
